# Herbal medicine for behavioral and psychological symptoms of dementia: A protocol for systematic review

**DOI:** 10.1097/MD.0000000000024577

**Published:** 2021-02-26

**Authors:** Chan-Young Kwon, Boram Lee, Da-Jung Ha

**Affiliations:** a Department of Oriental Neuropsychiatry, Dong-eui University College of Korean Medicine, 52-57, Yangjeong-ro, Busanjin-gu, Busan; b Clinical Medicine Division, Korea Institute of Oriental Medicine, 1672, Yuseong-daero, Yuseong-gu, Daejeon; c Department of Internal Medicine, Dong-eui University College of Korean Medicine, 52-57, Yangjeong-ro, Busanjin-gu, Busan, Republic of Korea.

**Keywords:** behavioral and psychological symptoms of dementia, dementia, herbal medicine, protocol, systematic review

## Abstract

**Background:**

Dementia is becoming a major public health problem worldwide with the aging of the world's population. Behavioral and psychological symptoms of dementia (BPSD), associated symptoms of dementia, not only predicts the poor prognosis of patients with dementia, but is also a major factor causing the care burden on caregivers, especially informal caregivers. For BPSD management, an alternative to existing psychotropic drugs is needed, given the benefit-harm ratio. Therefore, in this systematic review, we will evaluate the effectiveness and safety of herbal medicine for BPSD.

**Methods and analysis:**

Thirteen electronic databases will be comprehensively searched. Clinical studies reporting the efficacy (or effectiveness) and safety of herbal medicines in BSPD management published from their inception to December 2020 will be included. The primary outcome will be BPSD symptoms assessed by the validated tool. Moreover, total effective rate, daily living activities and quality of life of patients, burden and quality of life of caregiver, placement in a long-term care facility from home, and safety data will be regarded as the secondary outcome. Two independent researchers will perform the study selection, data extraction, and quality assessment process. To assess the methodological quality of the included studies, validated tools according to its design, such as the Cochrane Collaboration's risk of bias tool will be used. To perform meta-analysis, RevMan version 5.3 will be used, with mean differences for continuous outcomes and risk ratio for binary outcomes, and 95% confidence intervals. According to the heterogeneity and number of included studies, a fixed- or random-effects model will be used,

**Registration number:**

OSF (URL: https://osf.io/3u8ch), PROSPERO (CRD42020211000) (URL: https://www.crd.york.ac.uk/prospero/display_record.php?ID=CRD42020211000).

## Abstract 2

Supplemental Digital Content is available in the text

## 1 Introduction

Dementia is becoming a major public health problem worldwide with the aging of the world's population. The global economic and social burden associated with dementia is predicted to be USD 2 trillion by 2030, and the numbers continue to increase.^[1]^ The main clinical manifestation of dementia is cognitive decline, which is the core symptom of dementia.^[2]^ However, the development of treatments for this core symptom have experienced repeated failures, and accordingly, the best strategy for dementia today is prevention and early diagnosis.^[3]^ Some researchers argue that the multifactorial etiology of dementia may be explained by the failure to develop existing treatments for dementia.^[4]^

On the other hand, behavioral and psychological symptoms of dementia (BPSD) are associated symptoms of dementia.^[2]^ Although there are some differences in the prevalence depending on the type of dementia, most patients with dementia experience BPSD.^[5]^ BPSD does not only predicts a poor prognosis in patients with dementia but is also a major factor causing the care burden on caregivers, especially informal caregivers.^[6]^ In addition, BPSD is a predictor of early entry into facilities and can potentially contribute to the socioeconomic burden of dementia.^[6]^ Today, non-pharmacological treatments are primarily recommended for the management of BPSD. Though pharmacological approaches including psychotropic drugs are frequently being administered in clinical settings.^[7]^ However, the effects of psychotropic drugs on BPSD treatment are not satisfactory, and the most commonly used drugs, including antipsychotics, benzodiazepines, and Z-drugs, are associated with some fatal negative outcomes, such as an increased risk of falls and early death in elderly patients.^[8–10]^

In Asian countries, East Asian traditional medicine (EATM) modalities such as herbal medicine and acupuncture are widely used in health care and management of diseases. In particular, in countries such as Korea, China, and Japan, herbal medicines are managed at the national medical system level and can be prescribed by licensed experts, excluding some over the counter herbal products.^[11]^ Therefore, EATM modalities can be actively used to reduce the national economic and social burden of dementia in some countries.

Herbal medicine was previously reported to be effective on various neuropsychiatric diseases such as depression, anxiety, and insomnia.^[12]^ In addition, in the elderly, who make up the majority of patients with dementia, herbal medicine has been reported to have positive health promotion effects, such as anti-aging, improved quality of life (QoL), reduced senility, reduced sarcopenia, and prevented falls.^[13,14]^ In addition, unlike synthetic drugs, herbal medicine has the characteristics of multiple component-multiple target-multiple pathways, and thus may be a therapeutic approach suitable for a multifactorial etiology model of dementia.

Some existing systematic reviews have provided a review of representative anti-BPSD herbal medicines, such as Yokukansan.^[15]^ However, in clinical practice, various herbal medicines other than Yokukansan can be used,^[16]^ and a comprehensive review examining the role of herbal medicine for BPSD management in patients with dementia is necessary. Therefore, this protocol presents a plan for a comprehensive and critical systematic review of existing clinical studies reporting the efficacy (or effectiveness) and safety of herbal medicines in the management of BSPD.

## 2 Methods

### 2.1 Protocol registration

The protocol of this systematic review was registered in the OSF registries (URL: https://osf.io/3u8ch) and the International Prospective Register of Systematic Reviews (PROSPERO) (registration number, CRD42020211000) (URL: https://www.crd.york.ac.uk/prospero/display_record.php?ID=CRD42020211000). If protocol amendments occur, the dates, changes, and rationales will be tracked in PROSPERO. We reported this protocol according to the preferred reporting items for systematic review and meta-analysis protocols 2015 statement (Supplemental Digital Content 1).^[17]^

### 2.2 Data sources and search strategy

**Figure 1:**
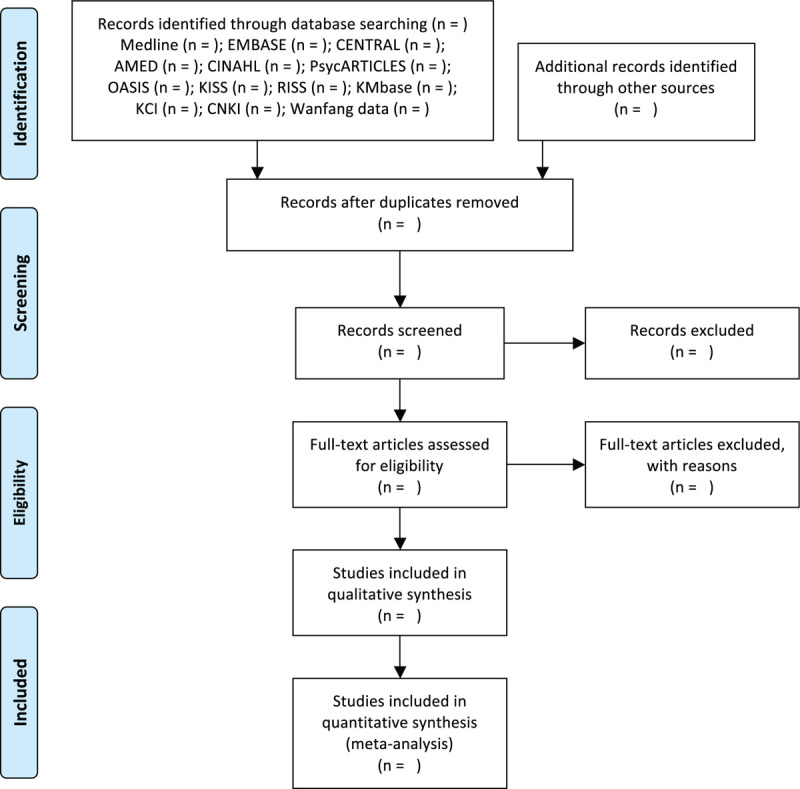
A PRISMA flow diagram of the literature screening and selection processes. AMED = Allied and Complementary Medicine Database, CENTRAL = Cochrane Central Register of Controlled Trials, CINAHL = Cumulative Index to Nursing and Allied Health Literature, CNKI = China National Knowledge Infrastructure, KCI = Korea Citation Index, KISS = Korean Studies Information Service System, KMbase = Korean Medical Database, OASIS = Oriental Medicine Advanced Searching Integrated System, RISS = Research Information Service System.

The following English, Korean, and Chinese electronic databases will be searched by 1 researcher (B Lee): Medline via PubMed, EMBASE via Elsevier, the Cochrane Central Register of Controlled Trials, Allied and Complementary Medicine Database via EBSCO, Cumulative Index to Nursing and Allied Health Literature via EBSCO, PsycARTICLES via ProQuest, Oriental Medicine Advanced Searching Integrated System, Korean Studies Information Service System, Research Information Service System, Korean Medical Database, Korea Citation Index, China National Knowledge Infrastructure, and Wanfang Data. Each electronic database will be searched for articles published from their inception to December 2020. We will identify additional articles to be included through reviews of relevant literature reference lists and trial registries such as clinicaltrials.gov, and consultation with experts in this area to include additional gray literature (Fig. 1). We described the search strategy used in Medline in Table 1.

**Table 1:** Search strategies for the Medline via PubMed.

#1. Dementia[MeSH] OR dement^∗^[Title/Abstract] OR Alzheimer^∗^[Title/Abstract] OR “Lewy body”[Title/Abstract] OR Huntington^∗^[Title/Abstract] OR Parkinson^∗^[Title/Abstract] OR “Pick disease”[Title/Abstract] OR “cognitive impairment”[Title/Abstract] #2. “Drugs, Chinese Herbal”[MH] OR “Medicine, Chinese Traditional”[MH] OR “Medicine, Kampo”[MH] OR “Medicine, Korean Traditional”[MH] OR “Korean medicine” OR “Chinese medicine” OR “Oriental medicine” OR “Kampo medicine” OR “herbal medicine” OR decoction^∗^ #3. #1 AND #2

### 2.3 Inclusion criteria

#### 2.3.1 Types of studies

Original clinical studies, including randomized controlled clinical trials, nonrandomized controlled clinical trials, and before-after studies to assess the beneficial effects and safety of herbal medicine on BPSD will be included. There will be no restrictions on publication language or publication status.

#### 2.3.2 Types of participants

Studies involving patients with any type of dementia in long-term care facilities, community, or specialized geriatric assessment and psychiatric units will be included. The diagnostic criteria will be the standardized diagnostic criteria, including the Diagnostic and Statistical Manual of Mental Disorders, the International Classification of Diseases, the National Institute of Neurological and Communicative Disorders, and Stroke and the Alzheimer Disease and Related Disorders Association or other recommended diagnostic criteria. There will be no restrictions on the gender, age, or race of the participants. However, studies that did not provide diagnostic criteria, or a validated assessment tool for inclusion, studies on patients with drug allergies, or other serious illnesses such as cancer, liver disease, or kidney disease will be excluded.

#### 2.3.3 Types of interventions

Studies involving oral herbal medicine as monotherapy or adjunctive therapies to psychotropic drugs such as anxiolytics, antidepressants, and antipsychotics, with or without routine care for dementia as treatment interventions will be included. Any dosage type of herbal medicine prescribed based on East Asian traditional medicine theories will be allowed. For control intervention, studies involving wait-list, placebo, or psychotropic drugs, such as anxiolytics, antidepressants, and antipsychotics, with or without routine care for dementia, will be included. Studies that do not list the compositions of the herbal medicines used will be excluded, except for patent drugs. Additionally, studies involving psychotherapy as treatment or control interventions will be excluded.

#### 2.3.4 Types of outcome measures

The primary outcome measures are as follows: the severity of BPSD symptoms, such as Behavior Pathology in Alzheimer Disease Rating Scale,^[18]^ Neuropsychiatric Inventory,^[19]^ Cohen-Mansfield Agitation Inventory,^[20]^ and Brief Psychiatric Rating Scale.^[21]^ The secondary outcome measures include (a) total effective rate for BPSD symptoms; (b) activities of daily living (ADL) of patients such as Barthel index,^[22]^ Katz index,^[23]^ and the functional independence measure,^[24]^ as well as instrumental activities of daily living such as activities of daily living prevention instrument,^[25]^ Alzheimer disease activities of daily living international scale,^[26]^ and Bayer activities of daily living scale^[27]^; (c) QoL of patients such as Alzheimer disease related quality of life,^[28]^ Dementia quality of life instrument,^[29]^ and quality of life in late-stage dementia scale^[30]^; (d) caregiver burden of caregiver such as caregiver burden inventory^[31]^; (e) QoL of caregiver such as short form 36 health survey^[32]^; (f) placement in long term care facility from home; and (g) safety data such as incidence of adverse events (AEs) and treatment discontinuation due to total or serious AEs.

### 2.4 Study selection

After reviewing the duplicate literature using an EndNote X8 (Clarivate Analytics, Philadelphia, PA), 2 researchers (CY Kwon and B Lee) will first review the possibility of inclusion by reviewing the title and abstract. After that, through full-text review of eligible studies, the final inclusion studies will be selected. Disagreements between the 2 researchers will be resolved through a consensus.

### 2.5 Data extraction

We will use a pre-defined and pilot-tested form of Excel 2016 (Microsoft, Redmond, WA) to extract data from the included studies. The extracted information will include the following: the first author's name, publication year, country, sample size and dropout, participants’ details, treatment intervention, comparison, duration of intervention, main outcome measures, AEs, and information for assessment of the risk of bias (RoB). Two researchers (CY Kwon and B Lee) will extract the data independently, and any disagreements will be resolved through a consensus. When the data in the included studies are insufficient, ambiguous, or missing, we will contact the corresponding authors of the original studies via e-mail.

### 2.6 Quality assessment

We will use the Cochrane Collaboration's RoB tool to assess the RoB of included randomized controlled clinical trials. Domains including random sequence generation, allocation concealment, blinding of participants, personnel, and outcome assessors, completeness of data outcome, selective reporting, and other biases will be assessed as “low risk,” “unclear risk,” or “high risk.”^[33]^ We will assess other bias items as the statistical baseline imbalance severity between the treatment and control groups, including the participant's mean age, sex, disease period, or disease severity. The RoB in nonrandomized studies of Interventions tool will be used to assess the RoB of included nonrandomized controlled clinical trials.^[34]^ The quality assessment tool for before-after (Pre-Post) studies with no control group, proposed by the National Heart, Lung, and Blood Institute, will be used to assess the RoB of included before-after studies.^[35]^ The quality assessment tool for case series studies, proposed by the National Heart, Lung, and Blood Institute, will be used to assess the RoB of included case reports/case series.^[35]^ Two researchers (CY Kwon and B Lee) will independently assess the methodological quality of included studies, and any discrepancies will be resolved through a consensus.

### 2.7 Data synthesis and analysis

Descriptive analysis of the findings, including the demographic characteristics of the participants, the details of the interventions, and the outcomes will be conducted for all included studies. Meta-analysis will be conducted for studies using the same type of interventions and comparators, with the same outcome measures, with Review Manager Software, version 5.4 (Cochrane, London, UK). Mean differences will be calculated for continuous outcomes, and the risk ratio will be calculated for binary outcomes, with 95% confidence intervals. We will assess heterogeneity using both the *χ*^2^ test and the *I*^2^ statistic, and we will consider an *I*^2^ value >50% as indicative of substantial heterogeneity, and an *I*^2^ value >75% as indicative of considerable heterogeneity. The results will be pooled using a random-effects model if included studies have significant heterogeneity (an *I*^2^ value >50%), while a fixed-effect model will be used if the heterogeneity is not significant, or if the number of studies included in the meta-analysis is very small (ie, less than 5), implying that the estimate of the between-study variance will lack precision.^[36,37]^

#### 2.7.1 Subgroup analysis

If necessary data are available, subgroup analyses will be conducted according to the following criteria:

(1) severity of dementia,
(2) type of dementia,
(3) severity of BPSD, and
(4) treatment duration.

#### 2.7.2 Sensitivity analysis

Sensitivity analyses to identify the robustness of the results of the meta-analysis will be conducted by excluding

(1) studies with high RoB and
(2) outliers that are numerically distant from the rest of the data.

#### 2.7.3 Assessment of reporting biases

If there are more than 10 studies included in the meta-analysis, a funnel plot will be used to assess publication bias.

### 2.8 Ethics and dissemination

Ethical approval will not be required since this is a systematic review. The results of this systematic review will be disseminated by the publication of a manuscript in a peer-reviewed journal or presentation at a relevant conference.

## 3 Discussion

Dementia is one of the major causes of the global economic and social burden.^[1]^ Associated symptoms of dementia or BPSD are associated with a poor prognosis in patients with dementia, burden on caregivers, early entry into facilities, and the resulting socioeconomic burden.^[6]^ For BPSD management, an alternative to existing psychotropic drugs is needed, given the benefit-harm ratio. In this context, the EATM strategy, which is actively used primarily in some Asian countries,^[11]^ can be an excellent medical resource. Among them, for herbal medicine, clinical evidence has already been reported for improving the health of the elderly as well as improving the effects on numerous psychiatric diseases through existing studies.^[12–14]^

This review will not be limited to specific herbal medicines such as Yokukansan, but will comprehensively collect and critically evaluate the anti-BPSD evidence of available herbal medicines in terms of EATM. The results of this review will not only help clinicians and patients establish an optimal dementia management strategy based on clinical evidence but also policy makers to devise strategies for alleviating the burden of dementia, a national task.

## Author contributions

**Conceptualization:** Chan-Young Kwon.

**Funding acquisition:** Chan-Young Kwon.

**Methodology:** Chan-Young Kwon, Boram Lee, Da-Jung Ha.

**Supervision:** Chan-Young Kwon.

**Writing – original draft:** Chan-Young Kwon.

**Writing – review and editing:** Chan-Young Kwon, Boram Lee.

## Supplementary Material

Supplemental Digital Content
